# Supplementary material for: Understanding fatality patterns and sex ratios of Brazilian free-tailed bats (Tadarida brasiliensis) at wind energy facilities in western California and Texas
Source: PeerJ. 2023 Dec 7;11:e16580. doi: 10.7717/peerj.16580 (PMC10710772; doi:10.7717/peerj.16580)
Supplement: Supplemental Information 2 — All roosts fall within the range of the two US migratory populations, and cave counts were available for at least one month during the peak population abundance at these roosts in the United States (June to August). We calculated the average proportion of females for each roost type by averaging the proportion of female fatalities at each site within the category. We did not use the raw counts of male and female bats at roost sites to determine the average proportion of females across roost types to avoid introducing biases from sites with larger bat populations. We calculated significance using a one-proportion z-test (p0 = 0.5, α = 0.05). Symbols: * = statistically significant male-skewed, ** = statistically significant female-skewed. [file peerj-11-16580-s002.docx]

**Supplemental Table 1. Estimates of the proportion of female Brazilian free-tailed bats at bridge roosts and cave roosts from the literature using morphological sex identification.**

All roosts fall within the range of the two US migratory populations, and cave counts were available for at least one month during the peak population abundance at these roosts in the United States (June to August). We calculated the average proportion of females for each roost type by averaging the proportion of female fatalities at each site within the category. We did not use the raw counts of male and female bats at roost sites to determine the average proportion of females across roost types to avoid introducing biases from sites with larger bat populations. We calculated significance using a one-proportion z-test (p_0_ = 0.5, α = 0.05). Symbols: * = statistically significant male-skewed, ** = statistically significant female-skewed.

| STATE | COUNTY | LOCATION | DATE | FEMALE COUNT | MALE COUNT | TOTAL | PROPO-RTION FEMALE | REFERENCE |
| --- | --- | --- | --- | --- | --- | --- | --- | --- |
| BRIDGE ROOST | | | | | | | | |
| OK | Grady | East Elm Creek Bridge | May–Oct. (2005) | 24 | 48 | 72 | 0.33* | Turmelle et al. (2010) |
| TX | Tom Green | Highway overpass at Foster Road and Highway Loop 306 | Mar.-Oct. (2014) | 442 | 319 | 763 | 0.58** | Martinez (2015) |
|  | Bell | Highway overpass at the intersection of Interstate 35 and Farm-to-Market Road (FM) 436 | Jun. –Nov. (1996) | 453 | 806 | 1259 | 0.36* | Sgro & Wilkins (2003) |
|  | Rusk | McNeail Bridge | May–Oct. (2005) | 54 | 27 | 81 | 0.67** | Turmelle et al. (2010) |
|  | Medina | Seco Creek Bridge | May–Oct. (2005) | 24 | 63 | 87 | 0.28* |  |
| Average Proportion of Females (Bridge Roost) | | | | | | | | **0.44** |

|  | | | | | | | | |
| --- | --- | --- | --- | --- | --- | --- | --- | --- |
| CAVE ROOST | | | | | | | | |
| NV | White Pine | Snake Range | Jun.–Aug. (2015) | 1,347 | 4,356 | 5,703 | 0.24* | Danielson et al. (2022) |
|  |  |  | Jun.–Oct. (2016) | 6,761 | 10,022 | 16,783 | 0.40* |  |
|  |  |  | Jul.–Sep. (2017) | 1,669 | 2,101 | 3,770 | 0.44* |  |
|  |  |  | Jun.–Oct. (2018) | 3,647 | 5,196 | 8,843 | 0.41* |  |
|  |  |  | Jul.–Oct. (2019) | 2,722 | 3,180 | 5,902 | 0.46* |  |
| OK | Major | Connor's Cave | Apr.–Sep. (1965) | 647 | 61 | 708 | 0.91** | Rogers (1972) |
|  |  |  | Apr.–Aug. (1966) | 306 | 48 | 354 | 0.87** |  |
|  |  |  | May–Aug. (1967) | 450 | 40 | 490 | 0.92** |  |
|  | Woods | Merrihew Cave | Apr.–Sep. (1965) | 340 | 176 | 516 | 0.66** |  |
|  |  |  | May–Aug. (1966) | 107 | 59 | 166 | 0.65** |  |
|  |  |  | May–Aug. (1967) | 194 | 121 | 315 | 0.61** |  |
|  |  |  | Apr.–Oct. (1952;1953) | 466 | 147 | 613 | 0.76** | Twente (1956) |
|  | Greer | Reed Cave | Jun.–Aug. (1965) | 371 | 38 | 409 | 0.91** | Rogers (1972) |
|  |  |  | Jun.–Aug. (1966) | 312 | 39 | 351 | 0.89** |  |
| OK | Greer | Reed Cave | Jun.–Aug. (1967) | 521 | 119 | 640 | 0.81** | Rogers (1972) |
|  | Woodward | Selman's Cave | Apr.–Oct. (1965) | 392 | 97 | 489 | 0.80** |  |
|  |  |  | May–Sep. (1966) | 280 | 55 | 335 | 0.84** |  |
|  |  |  | May–Sep. (1967) | 334 | 60 | 394 | 0.85** |  |
|  | Major | Vickery Cave | Apr.–Oct. (1965) | 586 | 123 | 709 | 0.83** | Rogers (1972) |
|  |  |  | May–Sep. (1966) | 242 | 64 | 306 | 0.80** |  |
|  |  |  | May–Sep. (1967) | 374 | 117 | 491 | 0.76** |  |
|  |  |  | May–Aug. (1990) | 50 | 20 | 70 | 0.71** | Thies (1993) |
|  |  |  | May–Aug. (1991) | 28 | 24 | 52 | 0.54 |  |
| TX | Comal | Braken Cave | Mar.–Aug. (1957) | 10,054 | 1,502 | 11,556 | 0.87** | Davis et al. (1962) |
|  |  |  | Jun., Sep. (1965) | 260 | 52 | 312 | 0.83** | Rogers (1972) |
|  |  |  | Jun., Sep. (1966) | 188 | 31 | 219 | 0.86** |  |
|  |  |  | Jun., Sep. (1967) | 154 | 53 | 207 | 0.74** |  |
|  | Blanco | Davis Cave | Mar.–Aug. (1957) | 7,962 | 1,516 | 9,478 | 0.84** | Davis et al. (1962) |
|  |  |  | Mar.–Sep. (1958) | 6,493 | 4,899 | 11,392 | 0.57** |  |
|  |  |  | Jun., Sep. (1965) | 375 | 72 | 447 | 0.84** | Rogers (1972) |
| TX | Blanco | Davis Cave | Jun., Sep. (1966) | 156 | 26 | 182 | 0.85** | Rogers (1972) |
|  |  |  | Jun., Sep. (1967) | 47 | 25 | 72 | 0.65** |  |
|  |  |  | May–Oct. (2005) | 63 | 7 | 70 | 0.90** | Turmelle et al. (2010) |
|  | Uvalde | Frio Cave | Jun., Sep. (1965) | 426 | 135 | 561 | 0.80** | Rogers (1972) |
|  |  |  | Jun., Sep. (1966) | 225 | 214 | 439 | 0.51 |  |
|  |  |  | Jun., Sep. (1967) | 140 | 70 | 210 | 0.67** |  |
|  |  |  | May–Oct. (2005) | 66 | 19 | 85 | 0.78** | Turmelle et al. (2010) |
|  | Mason | James River Cave | Mar.–Aug. (1957) | 7,591 | 2,141 | 9,732 | 0.85** | Davis et al. (1962) |
|  |  |  | May–Oct. (2005) | 78 | 3 | 81 | 0.96** | Turmelle et al. (2010) |
|  | Medina | Ney Cave | Jun.–Aug. (1949) | 472 | 208 | 680 | 0.69** | Cagle (1950) |
|  |  |  | Mar.–Aug. (1957) | 1,667 | 12,059 | 13,726 | 0.78** | Davis et al. (1962) |
| NM | Eddy | Carlsbad Caverns | May–Aug. (1991) | 27 | 24 | 51 | 0.53 | Thies (1993) |
| Average Proportion of Females (Cave Roosts) | | | | | | | | **0.73** |
